# Sorting signed circular permutations by super short operations

**DOI:** 10.1186/s13015-018-0131-6

**Published:** 2018-07-26

**Authors:** Andre R. Oliveira, Guillaume Fertin, Ulisses Dias, Zanoni Dias

**Affiliations:** 1 Institute of Computing, University of Campinas, Campinas, Brazil; 2 LS2N, UMR CNRS 6004, University of Nantes, Nantes, France; 3 School of Technology, University of Campinas, Limeira, Brazil

**Keywords:** Genome rearrangements, Super short operations, Circular permutations

## Abstract

**Background:**

One way to estimate the evolutionary distance between two given genomes is to determine the minimum number of large-scale mutations, or *genome rearrangements*, that are necessary to transform one into the other. In this context, genomes can be represented as ordered sequences of genes, each gene being represented by a signed integer. If no gene is repeated, genomes are thus modeled as signed permutations of the form $\pi =(\pi _1 \pi _2 \ldots \pi _n)$, and in that case we can consider without loss of generality that one of them is the identity permutation $\iota _n =(1 2 \ldots n)$, and that we just need to *sort* the other (i.e., transform it into $\iota _n$). The most studied genome rearrangement events are *reversals*, where a segment of the genome is reversed and reincorporated at the same location; and *transpositions*, where two consecutive segments are exchanged. Many variants, e.g., combining different types of (possibly constrained) rearrangements, have been proposed in the literature. One of them considers that the number of genes involved, in a reversal or a transposition, is never greater than two, which is known as the problem of sorting by *super short operations* (or SSOs).

**Results and conclusions:**

All problems considering SSOs in permutations have been shown to be in $\mathsf {P}$, except for one, namely sorting signed circular permutations by super short reversals and super short transpositions. Here we fill this gap by introducing a new graph structure called *cyclic permutation graph* and providing a series of intermediate results, which allows us to design a polynomial algorithm for sorting signed circular permutations by super short reversals and super short transpositions.

## Background

In bioinformatics, comparative genomics consists in analyzing the contents of two (or more) genomes in order to extract information. In particular, estimating the evolutionary distance between two extant species can be achieved by counting the minimum number of large-scale evolution events (called *genome rearrangements*) that separate two genomes. This is usually modeled as the following algorithmic problem: given two genomes $g_1$ and $g_2$ represented as ordered sequences of (possibly signed) genes, and a set $\mathcal {M}$ of allowed genome rearrangement events, determine the minimum number of events from $\mathcal {M}$ needed to obtain $g_2$ from $g_1.$

The first such genome rearrangement problems have been studied in the 1990s, and the topic has given rise to a very large literature since then (see, for example, Fertin et al. [1] for a survey). Two well-studied rearrangements are *reversals*, in which a segment of the genome is reversed and reincorporated at the same location, and *transpositions*, where two consecutive segments are exchanged.

If every gene appears exactly once in $g_1$ (resp. $g_2$), a genome can be represented by a (possibly signed) permutation, and one can without loss of generality rewrite the two input genomes $(g_1,g_2)$ into $(g'_1,g'_2)$, leaving the distance unchanged, and in such a way that $g'_2$ is the positive identity permutation $\iota _n$, where *n* is the number of genes in $g_1$ and $g_2$. In that case, we talk about *sorting* genome $g'_1.$

Sorting by reversals has been shown to be in $\mathsf {P}$ for signed genomes [2] and to be $\mathsf {NP}$-hard for unsigned genomes [3], where the best known approximation factor is 1.375 [4]. Sorting by transpositions is $\mathsf {NP}$-hard in unsigned genomes [5], and the best known approximation factor is 1.375 [6]. Sorting by reversals and transpositions is of unknown complexity both for signed and unsigned permutations, and the best known approximation factors is 2 for signed permutations [7] and 2*k* for unsigned permutations [8], where *k* is the approximation of the algorithm used for cycle decomposition [9].

Many other variants have been considered, notably considering different combinations and constraints for the set $\mathcal {M}$ of allowed rearrangements [1]. One of these variants considers that the number of genes involved in any rearrangement in $\mathcal {M}$ is never greater than two—such rearrangements are called *super short operations* (or SSOs). Although such models are of more theoretical interest, they are also motivated by the fact that rearrangements affecting large portions of a genome are less likely to occur [10] and that short reversals are prevalent in the evolution of some species [11, 12].

Sorting by SSOs has been studied in linear and circular genomes, signed and unsigned, when the allowed operations are reversals and/or transpositions. To cover circular genomes adequately, we will define in “Genome representation and super short operations” section *cyclic* SSOs, a particular type of SSOs which modify the permutation cyclically. On (a) unsigned permutations, we have that a super short reversal has the same effect of a super short transposition, which results in two different versions: (a.1) Sorting Permutations by SSOs and (a.2) Sorting Permutations by cyclic SSOs. Besides, as we will see in the next section, since transpositions cannot change the signs of elements, we do not use transpositions only on (b) signed permutations, so this operation must be used together with super short reversals. This results in four different problems: (b.1) Sorting Signed Permutations by Super Short Reversals, (b.2) Sorting Signed Permutations by cyclic Super Short Reversals, (b.3) Sorting Signed Permutations by SSOs, and (b.4) Sorting Signed Permutations by cyclic SSOs.

The summary of problems and the known results until now are shown in Table 1. In all cases the problem has been shown to be in $\mathsf {P}$, except for the latter case, which was left open, and which we solve in this paper. More precisely, we prove that sorting signed circular permutations by super short reversals and transpositions is in $\mathsf {P}$, thereby closing a gap in the literature concerning super short operations.

**Table 1:** List of sorting by SSOs and cyclic SSOs problems

Permutation type	Allowed genome rearrangement events	Polynomial-time algorithm
Unsigned	(a.1) Super short operations	[15]
Unsigned	(a.2) Cyclic super short operations	[13]
Signed	(b.1) Super short reversals	[16]
Signed	(b.2) Super short operations	[16]
Signed	(b.3) Cyclic super short reversals	[14]
Signed	(b.4) Cyclic super short operations	Here

This paper is organized as follows. “Preliminaries and notations” section presents some important concepts and notations that we use throughout the paper, notably the cp-graph that we introduce here and extensively use. “Related results” section presents a review on Sorting Permutations by SSOs and cyclic SSOs. In “Sorting Signed Permutations by cyclic SSOs” section, we provide a series of intermediate results, which allows us to design a polynomial algorithm for sorting signed linear permutations by *cyclic* super short reversals and transpositions. From this, we derive our main result, i.e., a proof that sorting signed circular permutations by super short reversals and transpositions is in $\mathsf {P}$. “Conclusion” section concludes the paper.

## Preliminaries and notations

In this section we present the important concepts and notations that we use throughout the paper.

### Genome representation and super short operations

A genome *g* can be transformed into a reduced mathematical representation by modeling it as an *n*-tuple whose elements represent its genes. In this paper, we assume that *g* contains no duplicated genes, thus the *n*-tuple is a permutation $\pi = (\pi _1 \pi _2 \ldots \pi _n)$, with $|\pi _i| \in \{1,2,\ldots,n\}$ and $|\pi _i| \ne |\pi _j|$ whenever $i \ne j$. Each element $\pi _i$ has a sign, $+$ or −, indicating the gene orientation, and we say that $\pi$ is a *signed* permutation. If the genome represented by $\pi$ is circular, then the elements $\pi _n$ and $\pi _1$ are considered to be adjacent and we say that $\pi$ is a *circular* permutation (also called *n-cycle* in the literature); otherwise $\pi$ is a *linear* permutation.

Given two permutations $\pi = (\pi _1 \pi _2 \ldots \pi _n)$ and $\sigma = (\sigma _1 \sigma _2 \ldots \sigma _n)$, the *composition* between $\pi$ and $\sigma$, denoted by $\pi \cdot \sigma$ results in the permutation $\alpha = (\alpha _1 \alpha _2 \ldots \alpha _n)$. If $\sigma _i < 0$, then $\alpha _i = {-\pi _{|\sigma _i|}}$, and $\alpha _i = \pi _{\sigma _i}$ otherwise. The *inverse* of $\sigma$, denoted by $\sigma ^{-1}$, is the permutation such that $\sigma ^{-1} \cdot \sigma = \iota _n$. That said, we can rewrite a pair of permutations $(\pi, \sigma )$ as $(\alpha, \iota _n)$, with $\alpha = \sigma ^{-1}\cdot \pi$, such that the distance between permutations $\pi$ and $\sigma$ is the same as the distance between permutations $\alpha$ and $\iota _n$. For example, if $\pi = (+\,1 - 3 + 2 + 5 - 4)$ and $\sigma = (+2 +4 -5 -1 +3)$, we have that $\sigma ^{-1} = (-\, 4 + 1 + 5 +2 -3)$, so the distance between permutations $\pi$ and $\sigma$ is the same as the distance between $\alpha = \sigma ^{-1}\cdot \pi = (-\, 4 -5 +1 -3 -2)$ and $\iota _5 = (+\,1 +2 +3 +4 +5).$

A *reversal* $\rho (i,j)$, $1 \le i \le j \le n$, is a rearrangement that reverses the order and signs of the genes in the subset of adjacent elements $\{\pi _i,...,\pi _j\}$. More precisely, it transforms the permutation $\pi$ into $\pi \cdot \rho (i, j) = (\pi _1 \ldots \underline{{-\pi }_j \ldots {-\pi }_i} \ldots \pi _n)$. A *cyclic reversal* $\rho ^*(i,j)$ is the extension of a reversal to the case where $i>j$: $\rho ^*(i,j)=\rho (i,j)$ if $i\le j$, whereas if $i > j$, then the subset reversed by $\rho ^*(i,j)$ is $\{\pi _i,...,\pi _n,\pi _1,...,\pi _j\}$. A reversal (resp. cyclic reversal) $\rho (i,j)$ (resp. $\rho ^*(i,j)$) is called a *z*-reversal, where $z = j-i+1 \pmod {n}$. We say that a *z*-reversal is *super short* if $z \in \{1,2\}.$

A *transposition* $\tau (i,j,k)$, $1 \le i< j < k \le n+1$, is a rearrangement that transforms $\pi$ into $\pi \cdot \tau (i,j,k) = (\pi _1 \ldots \pi _{i-1} \underline{\pi _j \ldots \pi _{k-1}} \; \underline{\pi _i \ldots \pi _{j-1}} \pi _{k} \ldots \pi _n)$. In other words, $\tau (i,j,k)$ exchanges subsets of adjacent elements $\{\pi _i,\ldots, \pi _{j-1}\}$ and $\{\pi _j,\ldots, \pi _{k-1}\}.$ Note that, since these subsets are not reversed, transpositions never change signs. As for reversals, a *cyclic transposition* $\tau ^*(i,j,k)$ is the extension of transpositions to the cases (a) $1 \le k< i < j \le n$ (in which subsets $\{\pi _i,\ldots, \pi _{j-1}\}$ and $\{\pi _j,\ldots, \pi _n,\pi _1,\ldots, \pi _{k-1}\}$ are exchanged), and (b) $1 \le j< k < i \le n$ (in which subsets $\{\pi _i,\ldots, \pi _n,\pi _1,\ldots, \pi _{j-1}\}$ and $\{\pi _j,\ldots, \pi _{k-1}\}$ are exchanged). A transposition (resp. cyclic transposition) $\tau (i,j,k)$ (resp. $\tau ^*(i,j,k)$) is called a *z*-transposition, where $z=x+y$ with $x = j-i \pmod {n}$ and $y = k-j \pmod {n}$, and we say that a *z*-transposition is *super short* if $z = 2.$

In the remainder of the paper, we will call a *super short operation* (or *SSO*) any 1-reversal, 2-reversal, or 2-transposition; moreover, any SSO that is not a 1-reversal will be called a *swap*, and the swap between elements $\pi _i$ and $\pi _j$ is denoted by $(\pi _i,\pi _j).$

Given a permutation $\pi$, the *sorting distance* of $\pi$, denoted by $d(\pi )$, is the length of a minimum-length sequence of SSOs needed to sort $\pi$. This paper is devoted to finding the smallest number of SSOs that are needed to sort a signed circular permutation $\pi$ of size *n* using for that a polynomial algorithm designed for sorting signed *linear* permutations by *cyclic* super short reversals and transpositions.

### VD-vector, crossing value, and crossing number

Most of the present paper will be concerned with *cyclic* SSOs in linear permutations. This section introduces a structure (called *valid displacement vector*) that allows us to compute the minimum number of cyclic swaps (i.e., 2-reversals or 2-transpositions) that put every element in its correct position (this number is called *crossing number*). It is important to note that this structure does not take into account the signs of the elements mainly for two reasons: (i) it does not make a distinction between 2-reversals and 2-transpositions (both are swaps), and (ii) it does not take into account 1-reversals, since they are not swaps by definition.

Given a sequence $\mathcal {S} = (s_1, s_2, \ldots, s_k)$ of *k* cyclic SSOs that sort a linear permutation $\pi$ ($\mathcal {S}$ is also called a *sorting sequence* for $\pi$), and given $1\le i\le n$, we denote by $R_\mathcal {S}(\pi _i)$ (resp. $L_\mathcal {S}(\pi _i)$) the number of cyclic SSOs in $\mathcal {S}$ that move $\pi _i$ to the right (resp. to the left).

For any $1\le i\le n$, the *displacement value* of $\pi _i$ with respect to $\mathcal {S}$ is given by $v_\mathcal {S}(\pi _i) = R_\mathcal {S}(\pi _i) - L_\mathcal {S}(\pi _i)$, and the *displacement vector* of $\pi$ associated to $\mathcal {S}$ is $V_\mathcal {S}(\pi ) = (v_\mathcal {S}(\pi _1), v_\mathcal {S}(\pi _2),\ldots, v_\mathcal {S}(\pi _n)).$

For instance, let us consider the permutation $\pi = (+\,4 +2 +3 -1 -5)$ and the sequence $\mathcal {S} = (\rho (3,4), \tau (2,3,4),\tau (1,2,3), \tau (2,3,4),\rho (3,4),\rho (4,4),\rho (5,5))$ of cyclic SSOs that sorts $\pi$. The sequence $\mathcal {S}$ results in the following sequence of swaps: $((+\,3,-\,1),(+\,2,+\,1), (+\,4,+\,1),(+\,4,+\,2),(+\,4,-\,3)).$ Note that $R_\mathcal {S}(-1) = 0$ and $L_\mathcal {S}(-1) = 3$, so $v_\mathcal {S}(-1) = R_\mathcal {S}(-1)-L_\mathcal {S}(-1)=0-3=-\,3$. One can also see that $V_\mathcal {S}(\pi ) = (3,0,0,-3,0).$

Let $X = (x_1, x_2, \ldots, x_n) \in \mathbb {Z}^n$ be a displacement vector and let $\pi$ be a permutation. We say that *X* is a *valid displacement vector* (or *VD-vector*) for $\pi$ if $\sum \nolimits _{i=1}^{n}{x_i} = 0$ (i.e., for each element of $\pi$ that moves one position to the right, another must move one position to the left and vice versa) and $|\pi _i| - x_i \equiv i \pmod {n}$ for $i \in [1..n]$ (i.e., every element must be in its correct position at the end). For instance, $X = (3,0,0,-3,0)$ is a VD-vector for $\pi = (+\,4 +2 +3 -1 -5)$ since $\sum \nolimits _{i=1}^{n}{x_i} = 3+0+0-3+0=0$ and $|\pi _i| - x_i \equiv i \pmod {n}$ for $i \in [1..5].$

Given a VD-vector $X = (x_1, x_2, \ldots, x_n) \in \mathbb {Z}^n$ and two distinct integers $i, j \in [1..n]$, let $r = i - j$ and $s = (i + x_i) - (j + x_j).$ Note that *r* measures how distant the element $\pi _j$ is from $\pi _i$ in $\pi$ (if $r > 0$ (resp. $r < 0$) then $\pi _j$ is located in a position to the left (resp. right) of $\pi _i$), while *s* measures how distant the element $\pi _j$ will be from $\pi _i$ in their final positions, i.e., positions $(i+x_i)$ for $\pi _i$ and $(j+x_j)$ for $\pi _j$. The *crossing value* between *i* and *j*, $1\le i \ne j\le n$, with respect to *X* is defined as follows:

$$\begin{aligned} c_{ij}(X) = {\left\{ \begin{array}{ll} |\{k \in [r..s] : k \equiv 0 \ (\mathrm {mod}\ n)\}|, \quad \text {if}\ r \le s;\\ -|\{k \in [s..r] : k \equiv 0 \ (\mathrm {mod}\ n)\}|, \quad \text {if}\ r > s. \end{array}\right. } \end{aligned}$$

In other words, $c_{ij}(X)$ represents the minimum number of times $\pi _i$ and $\pi _j$ are swapped (by a 2-reversal or a 2-transposition) in $\mathcal {S}_X$, a sorting sequence *X* is associated to. The sign of $c_{ij}(X)$ is positive (resp. negative) if $\pi _i$ is to the left (resp. right) of $\pi _j$ before the swap between these two elements takes place. For this reason, $c_{ii}(X)$ is undefined, and $c_{ij}(X)=-c_{ji}(X)$ for any $1\le i\ne j\le n$. Besides, $$x_i=\sum \nolimits _{\begin{subarray}{c} j = 1\\ j \ne i \end{subarray}}^{n} c_{ij}(X)$$ for $i \in [1..n].$

If *X* is a VD-vector associated to some sorting sequence for $\pi$, we say that *X* *induces* a swap $\alpha$ between two elements $\pi _i$ and $\pi _j$ if $i \ne j$ and $c_{ij}(x) \ne 0$. We also say that $\alpha$ is *induced* by *X*.

Given any VD-vector *X* for $\pi$, there exists at least one sorting sequence $\mathcal {S}$ such that $V_\mathcal {S}(\pi ) =X$. For instance, we can use 2-transpositions to apply the swaps induced by *X* (that will put every element in its correct position) followed by a sequence of 1-reversals applied to every negative element.

The *crossing number* of a VD-vector *X* is defined as $$cn(X) = \frac{1}{2}\sum \nolimits _{\begin{subarray}{c} i\ne {j} \end{subarray}}{|c_{ij}(X)|}$$. Informally, $cn(X)$ represents the minimum number of swaps in the sorting sequence *X* is associated to.

Take again $\pi = (+\,4 +2 +3 -1 -5)$ and the VD-vector $X = (3,0,0,-3,0)$. Given $i = 2$ and $j = 4$, we have that $r_1 = i-j = 2-4 = -2$ and $s_1 = (i+x_i)-(j+x_j) = 2-1 = 1$, and we have the crossing value $c_{24} = |\{k \in [-2..1] : k \equiv 0 \pmod {5}\}| = 1$. Now for $i = 3$ and $j = 1$, we have that $r_2 = i-j = 3-1 = 2$ and $s_2 = (i+x_i)-(j+x_j) = 3-4 = -1$, and we have $c_{31} = -|\{k \in [-1..2] : k \equiv 0 \pmod {5}\}| = -1$. After computing every crossing value, we obtain the crossing number $cn(X)=5.$

Given a VD-vector *X* for a permutation $\pi$, we denote by $d(\pi ,X)$ the size of a minimum-length sequence of SSOs needed to sort $\pi$ by applying swaps induced by *X*. Formally, $d(\pi ,X) = cn(X)+ y$, where *y* is the minimum number of 1-reversals over all sorting sequences having associated VD-vector *X*.

Given two integers $1\le i\ne j\le n$, we define the transformation $T_{i,j}(X) : \mathbb {Z}^n \rightarrow \mathbb {Z}^n$ over a VD-vector $X \in \mathbb {Z}^n$ as the one that creates the VD-vector $X'$ with $x_{k}' = x_k$, for $k \not \in \{i,j\}$, $x_i' = x_i - n$, and $x_j' = x_j + n.$

After such transformation is applied, each crossing value of the form $c_{ib}(X')$ and $c_{aj}(X')$ is one unit smaller than $c_{ib}(X)$ and $c_{aj}(X)$, respectively, and each crossing value of the form $c_{ai}(X')$ and $c_{jb}(X')$ is one unit larger than $c_{ai}(X)$ and $c_{jb}(X)$, respectively, with $1\le a,b \le n$, $a \not \in \{i,j\}$, and $b \not \in \{i,j\}$. Besides, $c_{ji}(X')$ (resp. $c_{ij}(X')$) is two units larger (resp. smaller) than $c_{ji}(X)$ (resp. $c_{ij}(X)).$

The following property was given by Jerrum [13]. Note that the author mistakenly wrote $cn(X') = cn(X) + 4(n-x_i+x_j)$, which was later corrected by Galvão et al. [14].

#### **Property 1**

*Let* $X \in \mathbb {Z}^n$ *be a VD-vector for* $\pi$ *, and let* $X' = T_{i,j}(X)$ *. Then* $cn(X') = cn(X) + 2(n-x_i+x_j).$

A transformation $T_{i,j}(X)=X'$ is called *contracting* (resp. *strictly contracting*) if and only if $x_i - x_j \ge n$ (resp. $x_i - x_j > n$), which implies by Property 1 that $cn(X') \le cn(X)$ (resp. $cn(X') < cn(X)$). If a VD-vector *X* admits no strictly contracting transformation, we have that $x_i - x_j \le n$ for any $1\le i\ne j\le n$, and thus for any VD-vector *Y*, $cn(Y)\ge cn(X).$

Given the VD-vector $X = (3,0,0,-3,0)$ for $\pi = (+\,4 +2 +3 -1 -5)$, we obtain the vector $X' = T_{1,4}(X)$ with $x'_1 = x_1-n = 3-5 = -2$ and $x'_4 = x_4+n = -3+5 = 2$, so $X' = (-\,2,0,0,2,0).$ Note that $X'$ is also a VD-vector for $\pi$, which means there exists a sorting sequence $\mathcal {S}'$ for $\pi$ such that $V_{\mathcal {S}'}(\pi )=X'$. By Property 1, $cn(X') = cn(X) +2(n - x_1 + x_4) =5-2 = 3.$

### Cyclic permutation graph

Since a VD-vector does not take into account the signs of the elements, we will introduce a new graph structure called *cyclic permutation graph*. This graph is constructed based on VD-vectors, and will help us to determine the minimum number of SSOs that sorts a permutation (now taking into account the signs of the elements and also considering 1-reversals).

Given a VD-vector *X* for a permutation $\pi$, we define the *cyclic permutation graph* (or cp-graph) of *X* and $\pi$, as the undirected graph $G^X_{\pi } = (V,E)$, with $V = \{\pi _1, \pi _2, \ldots , \pi _n\}$ and $E = \{\{\pi _i, \pi _j\} : c_{ij}(X) > 0\}.$

We associate weights to edges of $E(G^X_{\pi })$ as follows: if $e = \{\pi _i, \pi _j\}$ and $e \in E(G^{X}_{\pi })$, then $w(e) = c_{ij}(X)$. Note that, by construction, we have $\sum \nolimits _{e \in E(G^X_{\pi })} w(e)=cn(X)$. If every edge $e \in E(G^X_{\pi })$ satisfies $w(e) =1$, then for any $i \in [1..n]$ vertex $\pi _i$ has at least $|x_i|$ edges (since $x_i=\sum _{j=1}^{n} c_{ij}(X)$), and thus the connected component that contains $\pi _i$ has at least $|x_i| + 1$ vertices.

We denote by $cc(G^X_{\pi })$ the number of connected components of $G^X_{\pi }$. Moreover, a connected component of $G^X_{\pi }$ is said to be *odd* if it contains an odd number of vertices $\pi _i$ such that $\pi _i<0$, and is said to be *even* otherwise. The number of odd connected components in $G^X_{\pi }$ is denoted $cc^-(G^X_{\pi }).$

Let $\pi$ be a permutation, $X = (x_1, x_2, \ldots , x_n) \in \mathbb {Z}^n$ be a VD-vector for $\pi$, and $\alpha$ be a cyclic SSO induced by *X* (i.e., $\alpha$ is a 2-reversal or a 2-transposition) applied to two adjacent elements $\pi _i$ and $\pi _{i\pmod {n}+1}$ of $\pi$. The resulting VD-vector $X'$ for $\pi ' = \pi \cdot \alpha$ is such that $x'_k = x_k$ for every $k \not \in \{i,{i\pmod {n}+1}\}$, $x'_i = x_{i\pmod {n}+1}+1$ and $x'_{i\pmod {n}+1} = x_i -1$. Moreover, $cn(X') = cn(X) - 1$, and the cp-graph $G^{X'}_{\pi '}$ can be obtained from $G^X_{\pi }$ by decreasing the weight of the edge between vertices $\pi _i$ and $\pi _{i\pmod {n}+1}$ by 1 (or by removing that edge if its previous weight was one).

For instance, take again $\pi = (+\,4 +2 +3 -1 -5)$, $X = (3, 0, 0, {-3}, 0)$, and $X' = T_{1,4}(X) = (-\,2, 0, 0, 2, 0)$. We can apply the 2-reversal $\rho (4,5)$ that is induced by $X'$ to obtain the permutation $\pi ' = \pi \cdot \rho (4,5) = (+\,4 +2 +3 +5 +1)$ and the VD-vector $X'' = ({-2}, 0, 0, 1, 1)$.

**Fig. 1:**
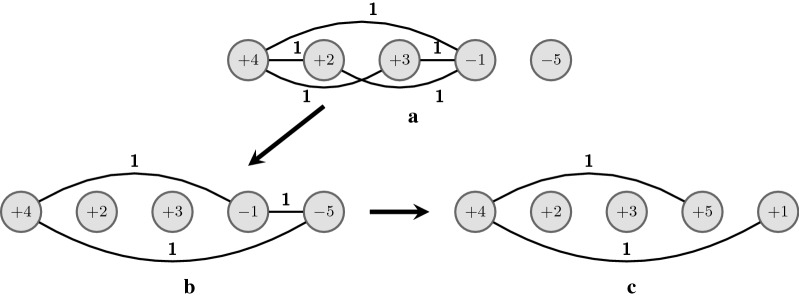
In **a** we have the cp-graph $G^{X}_{\pi }$ for $\pi = (+\,4 +2 +3 -1 -5)$ and $X = (3, 0, 0, {-3}, 0)$, with $cn(X) = 5$, $cc(G^{X}_{\pi }) = 2$, and $cc^-(G^{X}_{\pi }) = 2$. In **b** we have the cp-graph $G^{X'}_{\pi }$ for $X' = T_{1,4}(X) = (-\,2, 0, 0, 2, 0)$. By Property 1 $cn(X') = cn(X) + 2(n - x_1 + x_4) = 5-2 = 3$ (recall that $cn(X')$ is also the sum of weights in the graph $G^{X'}_{\pi }$), $cc(G^{X'}_{\pi }) = 3$, and $cc^-(G^{X'}_{\pi }) = 0$. We can see in $G^{X}_{\pi }$ and $G^{X'}_{\pi }$ that the 2-reversal $\rho (4,5)$ is induced by $X'$ but not by *X*; **c** the cp-graph $G^{X''}_{\pi '}$ for $\pi ' = \pi \cdot \rho (4,5) = (+\,4 +2 +3 +5 +1)$ and its VD-vector $X'' = ({-2}, 0, 0, 1, 1)$ with $cn(X'') = cn(X')-1 = 2$, $cc(G^{X''}_{\pi '}) = 3$, and $cc^-(G^{X''}_{\pi '}) = 0$

The corresponding cp-graphs $G^{X}_{\pi }$, $G^{X'}_{\pi }$, and $G^{X''}_{\pi '}$ are given in Fig. 1. In Fig. 1a we have $cn(X) = 5$ and $cc(G^{X}_{\pi }) = cc^-(G^{X}_{\pi }) = 2$; in Fig. 1b we have $cn(X') = 3$, $cc(G^{X'}_{\pi }) = 3$, and $cc^-(G^{X'}_{\pi })=0$; in Fig. 1c we have $cn(X'') = 2$, $cc(G^{X''}_{\pi '}) = 3$, and $cc^-(G^{X''}_{\pi '})=0$.

## Related results

In this section, we provide related results of solving Sorting Permutations by SSOs and cyclic SSOs problems.

### Sorting by SSOs

Given a permutation $\pi$, a pair of elements $(\pi _i,\pi _j)$ is called an *inversion* if $|\pi _i| > |\pi _j|$ and $i < j$, with $i \ne j$ and $\{i,j\} \in [1..n]$. Let $inv(\pi )$ be the number of inversions in $\pi$. Knuth [15, p. 108] showed in 1973 that Sorting Unsigned Permutations by SSOs belongs to $\mathsf {P}$ and that the sorting distance for this version is $d(\pi ) = inv(\pi )$. For instance, taking the unsigned permutation $\alpha = (6 4 2 3 1 5 7)$, we have that $inv(\alpha ) = |\{(6,4),(6,2),(6,3),(6,1),(6,5),(4,2),(4,3),$ $(4,1),(2,1),(3,1)\}| = 10$ so it follows that $d(\alpha ) = 10$.

The number of inversions is a natural lower bound for Sorting Signed Permutations by SSOs: for any signed permutation $\pi$, $d(\pi ) \ge inv(\pi )$. Galvão et al. [16] proved in 2015 that Sorting Signed Permutations by Super Short Operations is in $\mathsf {P}$. Let $IG(\pi )$ be the *inversion graph* of the signed permutation $\pi$. $IG(\pi )$ is such that $V(IG(\pi ))$ is formed by the elements of $\pi$ and $E(IG(\pi ))$ is formed by the pairs of inversions in $\pi$. A component in $IG(\pi )$ is *odd* if it contains an odd number of negative elements, and it is *even* otherwise. The authors showed that there exists a minimum sorting sequence for this problem that uses $inv(\pi )$ swaps plus *k* 1-reversals, such that *k* is the number of odd components in $IG(\pi )$. For instance, taking the signed permutation $\alpha ' = (-\,6 +4 +2 -3 +1 +5 -7)$, we have that $IG(\alpha ')$ has two components: an odd component with the element ${-7}$ only, and an even component with the remaining elements, so it follows that $d(\alpha ') = 10 + 1 = 11$. Note that the sorting distance for $\alpha '$ is decreased by two compared to the version that allows only super short reversals.

### Sorting by cyclic SSOs

Jerrum [13] showed in 1985 that Sorting Unsigned Permutations by cyclic SSOs belongs to $\mathsf {P}$. The author proved that the sorting distance for this version is $d(\pi ) = \min \{cn(X) : X$ is a VD-vector for $\pi \}$. Take the permutation $\alpha = (6 4 2 3 1 5 7)$ again. We have that $X = (-\, 2,2,-1,-1,3,-1,0)$, with $cn(X) = 6$, is a VD-vector for $\alpha$. Besides, for any VD-vector $X'$ for $\alpha$, $cn(X') \ge cn(X)$, so it follows that $d(\alpha ) = 6$. Note that the sorting distance for $\alpha$ decreases from 10 to 6 by allowing cyclic SSOs.

In 2016, Galvão et al. [14] proved that Sorting Signed Permutations by cyclic super short reversals is also in $\mathsf {P}$. Given a signed permutation $\pi$ and a VD-vector *X*, let $neven(X,\pi )$ be the set of elements from $\pi$ such that $|x_i|$ is even and $\pi _i < 0$, and let $podd(X,\pi )$ be the set of elements from $\pi$ such that $|x_i|$ is odd and $\pi _i > 0$. In a similar way as in Sorting Signed Permutations by SSOs, the authors proved that, given any VD-vector *X* for $\pi$ such that *X* has the minimum crossing number over all VD-vectors for $\pi$, the sorting distance of $\pi$ is precisely $cn(X) + k$, where *k* is the number of elements in $\{neven(X,\pi ) \cup podd(X,\pi )\}$. Taking $\alpha ' = (-\, 6 +4 +2 -3 +1 +5 -7)$ and $X = (-\, 2,2,-1,-1,3,-1,0)$ (recall that any VD-vector $X'$ for $\alpha '$ is such that $cn(X') \ge cn(X)$), we have $cn(X) = 6$, $neven(X,\alpha ') = \{{-6},{-7}\}$, and $podd(X,\alpha ') = \{{+1},{+2},{+5}\}$, so it follows that $d(\alpha ') = 6+5 = 11$. Compared to the version that does not allow cyclic super short reversals, the sorting distance for $\alpha '$ decreases from 13 to 11.

For Sorting Signed Permutations by cyclic SSOs, a trivial lower bound comes from the unsigned version with cyclic SSOs: $d(\pi ) \ge \min \{cn(X) : X$ is a VD-vector for $\pi \}$. Inspired by $IG(\pi )$, we defined in “Cyclic permutation graph” section the cp-graph, creating edges according to the crossing values of a VD-vector *X* instead of inversions. Although these graphs are different, the classification of odd and even components is the same and will be useful later.

Note that in all previous problems showed in this section the sorting distance is always associated with the minimum number of inversions or the minimum crossing number. What makes Sorting Signed Permutations by cyclic SSOs not trivial is that, as we will see later, unlike all previous problems a minimum sorting sequence is not necessarily associated to a VD-vector with minimum crossing number (see Fig. 5 for an example).

### Sorting linear permutations by cyclic SSOs vs. sorting circular permutations by SSOs

Note that, although sorting linear permutations by cyclic SSOs and sorting circular permutations by SSOs are different problems, we can use the first to solve the latter. Just as an example, the permutation $\pi = (+\,5 +4 -2 -1 +3)$ has a sorting distance of 8, considering the model that only allows SSOs, and it has a sorting distance of 4 considering the model that allows cyclic SSOs. But if $\pi$ is circular, then $\pi ' = (-\, 2 -1 +3 +5 +4)$ is also a linear representation for $\pi$, since it respects all adjacencies between elements. This linear representation $\pi '$ has a sorting distance of 2 for the model that only allows SSOs and also in the model that allows cyclic SSOs. Besides, $\pi '$ is, in fact, the linear representation for the circular permutation $\pi$ with the lowest sorting distance by cyclic SSOs, so it follows that the sorting distance of the circular permutation $\pi$ is 2. A more detailed explanation of how to use a linear model to solve circular permutations will be given at the end of “Sorting Signed Permutations by cyclic SSOs” section.

## Sorting Signed Permutations by cyclic SSOs

This section is devoted to proving our two main results, namely the fact that sorting signed linear permutations by *cyclic* SSOs is in $\mathsf {P}$ (Theorem 1), and, consequently, that sorting signed circular permutations by SSOs is also in $\mathsf {P}$ (Theorem 2). For this, we study in depth (and provide properties of) sorting signed linear permutations by cyclic SSOs, which heavily rely on the cp-graph we introduced in “Preliminaries and notations” section.

### Properties of VD-vectors

Before we provide a series of lemmas that will lead to our final algorithm, we begin with the three following properties, which will prove useful in this section.

In Property 2 we will show that if a VD-vector *X* has a displacement value $x_i$ whose absolute value is greater than or equal to *n*, then there is a crossing value $c_{ij}(X)$ (in absolute value) greater than one. In Property 3 we will show that if a VD-vector *X* has a crossing value $c_{ij}(X)$ greater than zero, then elements $\pi _k$, $k \in [i..j]$, must be in the same component in its corresponding cp-graph. Property 4 is an extension of Property 3, where we show that if a VD-vector *X* has a crossing value $c_{ij}(X)$ (in absolute value) greater than one, then all elements are in the same component in its corresponding cp-graph.

#### **Property 2**

*Let* $X = (x_1, x_2, ..., x_n) \in \mathbb {Z}^n$ *be a VD-vector for* $\pi$ *. If there exists* $1\le i\le n$ *such that* $|x_i| \ge n$ *, then there exists* $1\le j\ne i\le n$ *such that* $|c_{ij}(X)| > 1.$

#### *Proof*

Let $X \in \mathbb {Z}^n$ be a VD-vector for $\pi$, and let us suppose that there exists $1\le i\le n$ such that $|x_i| \ge n$. We know, by definition, that $x_i=\sum _{j=1, j\ne i}^{n} c_{ij}(X)$. Since there are $n-1$ crossing values of the form $c_{ij}(X)$ (one for each $j\ne i$), this necessarily implies that $|c_{ij}(X)| > 1$ for some $1\le j\ne i\le n$. $\square$

#### **Property 3**

*Let* $X = (x_1, x_2, ..., x_n) \in \mathbb {Z}^n$ *be a VD-vector for* $\pi$ *. If* $c_{ij}(X) > 0$ *(resp.* $c_{ij}(X) < 0$ *) for some* $1\le i\ne j\le n$ *, then, for any* $k \in [i+1..j-1]$ *(resp.* $k \in [j+1..i-1]$ *), we have* $|c_{ik}(X)| + |c_{jk}(X)| \ne 0$ *and* $\{\{\pi _i,\pi _k\},\{\pi _j,\pi _k\}\} \cap E(G^X_{\pi }) \ne \emptyset.$

#### *Proof*

Let *X* be a VD-vector such that for two elements $\pi _i$ and $\pi _j$, $1\le i\ne j\le n$, $c_{ij}(X) \ne 0$. Since $c_{ij}(X) = -c_{ji}(X)$, let us suppose, without loss of generality, that $c_{ij}(X) = \gamma$ with $\gamma \ge 1$. Let $r_1 = i - j$ and $s_1 = (i + x_i) - (j + x_j)$. Since $c_{ij}(X)$ is positive we have, by definition of crossing value, that $r_1 \le s_1$.

Suppose first that $i < j$. Since $r_1 = i - j < 0$ and $r_1 \le s_1$, then $s_1 > \gamma -1$, otherwise $c_{ij}(X) < \gamma$. We have that $x_i > x_j +j -i + \gamma - 1$. Suppose that we have an element $\pi _k$ with $i< k < j$ such that $c_{ik}(X) = c_{jk}(X) = 0$. For $c_{ik}(X)$, we have that $r_2 = i - k < 0$, so $s_2 = (i + x_i) - (k + x_k) < 0$, otherwise $c_{ik}(X) \ne 0$. It follows that $x_k > x_i + i - k$, and, since $x_i > x_j +j -i + \gamma - 1$, we have that $x_k > x_j +j -k + \gamma - 1$. For $c_{jk}(X)$, we have that $r_3 = j - k > 0$, so $s_3 = (j + x_j) - (k + x_k) > 0$, otherwise $c_{jk}(X) \ne 0$. It follows that $x_k < x_j + j - k$, which is a contradiction to the fact that $x_k > x_j +j -k + \gamma - 1$ and $\gamma > 0$, so we conclude that $|c_{ik}(X)| + |c_{jk}(X)| \ne 0$.

Now let us suppose $i > j$. In this case, we can split the interval that goes from *i* to *j* into $[i+1..n] \cup [1..j-1]$. Since $r_1 = i - j > 0$ and $r_1 \le s_1$, then $s_1 \ge \gamma {n}$, otherwise $c_{ij}(X) < \gamma$. It follows that $x_i \ge x_j +j -i +\gamma {n}$. Suppose that we have an element $\pi _k$ such that $k \in [i+1..n] \cup [1..j-1]$ and $c_{ik}(X) = c_{jk}(X) = 0$. We have to consider two cases: when $k \in [1..j-1]$ (i.e., $k < \min (i,j)$) and when $k \in [i+1..n]$ (i.e., $k > \max (i,j)$):

1. $k < \min (i,j)$: in this case, for both $c_{ik}(X)$ and $c_{jk}(X)$, we have that $r_2 = i - k > 0$ and $r_3 = j - k > 0$, so $0< s_2 < n$ and $0< s_3 < n$, otherwise $|c_{ik}(X)| + |c_{jk}(X)| \ne 0$. For $c_{ik}(X)$ and $s_2 = (i + x_i) - (k + x_k) < n$, we have $x_i + i - x_k - k < n$, resulting in $x_k > x_i + i -k -n$. Since $x_i \ge x_j +j -i +\gamma {n}$, we have that $x_k > x_j + j -k+(\gamma -1)n$. For $c_{jk}(X)$ and $s_3 = (j + x_j) - (k + x_k) > 0$, we have $x_j + j - x_k - k > 0$, resulting in $x_k < x_j +j -k$, which is a contradiction to the fact that $x_k > x_j + j -k +(\gamma -1)n$ and $(\gamma -1) \ge 0$.
2. $k > \max (i,j)$: in this case, for both $c_{ik}(X)$ and $c_{jk}(X)$, we have that $r_2 = i - k < 0$ and $r_3 = j - k < 0$, so $-n< s_2 < 0$ and $-n< s_3 < 0$, otherwise $|c_{ik}(X)| + |c_{jk}(X)| \ne 0$. For $c_{ik}(X)$ and $s_2 = (i + x_i) - (k + x_k) < 0$, we have $x_i + i - x_k - k < 0$, resulting in $x_k > x_i + i -k$. Since $x_i \ge x_j +j -i +\gamma {n}$, we have that $x_k > x_j + j -k +\gamma {n}$. For $c_{jk}(X)$ and $s_3 = (j + x_j) - (k + x_k) > -n$, we have $x_j + j - x_k - k > -n$, resulting in $x_k < x_j +j -k +n$, which is a contradiction to the fact that $x_k > x_j + j -k +\gamma {n}$ and $\gamma \ge 1$.

In all cases, it follows that $|c_{ik}(X)| + |c_{jk}(X)| \ne 0$, thus $\{\{\pi _i,\pi _k\},\{\pi _j,\pi _k\}\} \cap E(G^X_{\pi }) \ne \emptyset$. $\square$

#### **Property 4**

*Let* $X = (x_1, x_2, ..., x_n) \in \mathbb {Z}^n$ *be a VD-vector for* $\pi$ *. If there exists a* $c_{ij}(X)$ *such that* $|c_{ij}(X)| > 1$ *then* $cc(G^X_{\pi })=1.$

#### *Proof*

Let *X* be a VD-vector for some permutation $\pi$ such that $|c_{ij}(X)|> 1$ for some $1\le i\ne j\le n$. Let us suppose, without loss of generality, that $c_{ij}(X)>1$ (recall that $c_{ij}(X)= -c_{ji}(X)$ by definition), and for readability, let $c_{ij}(X)=\gamma$. Let $r_1 = i - j$ and $s_1 = (i + x_i) - (j + x_j)$. Since $c_{ij}(X)$ is positive we have, by definition of crossing value, that $r_1 \le s_1$. Let us now consider two cases: either $i < j$, or $i > j$.

Suppose first $i < j$. In this case, $r_1 = i - j$ is such that ${-n}< r_1 < 0$. Since $r_1 \le s_1$, we have that $s_1 = (i + x_i) - (j + x_j) \ge (\gamma -1)n$ (we use ($\gamma -1$) because $\gamma = |\{k \in [r_1..s_1] : k \equiv 0 \pmod {n}\}| = |\{k \in [r_1..0] : k \equiv 0 \pmod {n}\}| + |\{k \in [1..s_1] : k \equiv 0 \pmod {n}\}| = 1 + |\{k \in [1..s_1] : k \equiv 0 \pmod {n}\}|$, so $(\gamma - 1) = |\{k \in [1..s_1] : k \equiv 0 \pmod {n}\}|$ and $s_1 \ge (\gamma -1)n$), otherwise $c_{ij}(X) < \gamma$. It follows that $x_i \ge (\gamma -1)n + x_j +j -i$. Now suppose that we have an element $\pi _k$ such that $c_{ik}(X) = c_{jk}(X) = 0$. We have three cases:

1. $k< i < j$: in this case, $r_2 = i - k > 0$ and $r_3 = j - k > 0$, so we must have that $0< s_2 < n$ and $0< s_3 < n$, with $s_2 = (i + x_i) - (k + x_k)$ and $s_3 = (j + x_j) - (k + x_k)$. For $s_2 < n$, we have $x_k > x_i + i -k -n$, and since $x_i \ge (\gamma -1)n + x_j +j -i$ we have that $x_k > (\gamma -2)n +x_j +j -k$. For $s_3 > 0$, we have $x_k < x_j + j -k$, but this is not possible since $(\gamma -2)n \ge 0$.
2. $i< k < j$: in this case, $r_2 = i - k < 0$ and $r_3 = j - k > 0$, so $-n< s_2 < 0$ and $0< s_3 < n$, with $s_2 = (i + x_i) - (k + x_k)$ and $s_3 = (j + x_j) - (k + x_k)$. For $s_2 < 0$, we have $x_k > x_i + i -k$, and since $x_i \ge (\gamma -1)n + x_j +j -i$ we have that $x_k > (\gamma -1)n +x_j +j -k$. For $s_3 > 0$, we have $x_k < x_j + j -k$, but this is not possible since $(\gamma -1)n > 0$.
3. $i< j < k$: in this case, $r_2 = i - k < 0$ and $r_3 = j - k < 0$, so $-n< s_2 < 0$ and $-n< s_3 < 0$, with $s_2 = (i + x_i) - (k + x_k)$ and $s_3 = (j + x_j) - (k + x_k)$. For $s_2 < 0$, we have $x_k > x_i + i -k$, and since $x_i \ge (\gamma -1)n + x_j +j -i$ we have that $x_k > (\gamma -1)n +x_j +j -k$. For $s_3 > -n$, we have $x_k < x_j + j -k+n$, but this is not possible since $(\gamma -1)n \ge n$.

Now let us consider $i > j$. In this case, $r_1 = i - j$ is such that $0< r_1 < n$. Since $r_1 \le s_1$, we have that $s_1 = (i + x_i) - (j + x_j) \ge \gamma {n}$, otherwise $c_{ij}(X) < \gamma$. It follows that $x_i \ge \gamma {n} + x_j +j -i$. Now suppose that we have an element $\pi _k$ such that $c_{ik}(X) = c_{jk}(X) = 0$. We also have three cases:

1. $k< j < i$: in this case, $r_2 = i - k > 0$ and $r_3 = j - k > 0$, so we must have that $0< s_2 < n$ and $0< s_3 < n$, with $s_2 = (i + x_i) - (k + x_k)$ and $s_3 = (j + x_j) - (k + x_k)$. For $s_2 < n$, we have $x_k > x_i + i -k -n$, and, since $x_i \ge \gamma {n} + x_j +j -i$, we have that $x_k > (\gamma -1)n +x_j +j -k$. For $s_3 > 0$, we have $x_k < x_j + j -k$, but this is not possible since $(\gamma -1)n > 0$.
2. $j< k < i$: in this case, $r_2 = i - k > 0$ and $r_3 = j - k < 0$, so $0< s_2 < n$ and $-n< s_3 < 0$, with $s_2 = (i + x_i) - (k + x_k)$ and $s_3 = (j + x_j) - (k + x_k)$. For $s_2 < n$, we have $x_k > x_i + i -k -n$, and, since $x_i \ge \gamma {n} + x_j +j -i$, we have that $x_k > (\gamma -1)n +x_j +j -k$. For $s_3 > -n$, we have $x_k < x_j + j -k+n$, but this is not possible since $(\gamma -1)n \ge n$.
3. $j< i < k$: in this case, $r_2 = i - k < 0$ and $r_3 = j - k < 0$, so $-n< s_2 < 0$ and $-n< s_3 < 0$, with $s_2 = (i + x_i) - (k + x_k)$ and $s_3 = (j + x_j) - (k + x_k)$. For $s_2 < 0$, we have $x_k > x_i + i -k$, and, since $x_i \ge \gamma {n} + x_j +j -i$, we have that $x_k > \gamma {n} +x_j +j -k$. For $s_3 > -n$, we have $x_k < x_j + j -k+n$, but this is not possible since $\gamma {n} > n$.

It follows that if $c_{ij}(X) > 1$, then, for any element $\pi _k$ with $k \not \in \{i,j\}$, we have that $|c_{ik}(X)| + |c_{jk}(X)| \ne 0$, so $\{\{\pi _i,\pi _k\},\{\pi _j,\pi _k\}\} \cap E(G^X_{\pi }) \ne \emptyset$. Since $c_{ij}(X) > 1$, we also have that $\{\pi _i,\pi _j\}\in G^X_{\pi }$, so $cc(G^X_{\pi })=1$. $\square$

### SSOs and the cp-graphs

In this section we provide five lemmas relating SSOs with cp-graphs. In Lemma 1 (resp. Lemma 2) we will analyze the difference in the number of odd components in the cp-graph when we apply a 1-reversal (resp. a swap, i.e., a 2-reversal or a 2-transposition) to the permutation $\pi$. In Lemma 3 we will show that we can always apply a swap induced by *X* without increasing the number of odd components in the resulting cp-graph. Let $|\mathcal {S}|$ denote the length of a sorting sequence $\mathcal {S}$. In Lemma 4 (resp. Lemma 5) we will show that if a sorting sequence $\mathcal {S}$ has an SSO that increases the number of odd components (resp. the weight of an edge), then there is another sorting sequence $\mathcal {S'}$ with $|\mathcal {S'}| \le |\mathcal {S}|$ such that $\mathcal {S'}$ does not contain such SSOs.

#### **Lemma 1**

*Let X and* $X'$ *be two VD-vectors of* $\mathbb {Z}^n$ *such that X is a VD-vector for* $\pi$ *and* $X'$ *is a VD-vector for* $\pi ' = \pi \cdot \alpha$ *, where* $\alpha$ *is a cyclic SSO. If* $\alpha$ *is a 1-reversal, then* $X' = X,$ $cn(X') = cn(X)$ *, and* $\Delta {cc^-} = cc^-(G^{X'}_{\pi '}) - cc^-(G^X_{\pi }) \in \{-1, 1\}.$

#### *Proof*

If $\alpha$ is a 1-reversal, we have that $|\pi '_i| = |\pi _i|$ for every $1 \le i \le n$, which implies that $X' = X$, $cn(X') = cn(X)$, and $cc(G^{X'}_{\pi '}) = cc(G^X_{\pi })$. Now if the connected component impacted by $\alpha$ in $G^X_{\pi }$ is even (resp. odd), then it will become odd (resp. even) in $G^{X'}_{\pi '}$, thus $\Delta {cc^-} \in \{-1, 1\}$. $\square$

#### **Lemma 2**

*Let X and* $X'$ *be two VD-vectors of* $\mathbb {Z}^n$ *such that X is a VD-vector for* $\pi$* and* $X'$ *is a VD-vector for* $\pi ' = \pi \cdot \alpha$*, where* $\alpha$ *is a cyclic SSO. If* $\alpha$ *is a 2-reversal or a 2-transposition induced by X in* $\pi$*, then* $cn(X') = cn(X)-1$ *and either* $cc(G^{X'}_{\pi '}) = cc(G^X_{\pi })$* and* $\Delta {cc^-} = cc^-(G^{X'}_{\pi '}) - cc^-(G^X_{\pi }) = 0$* or *$cc(G^{X'}_{\pi '}) > cc(G^X_{\pi })$* and* $\Delta {cc^-} \in \{0,2\}$.

#### *Proof*

Since $\alpha$ is a cyclic SSO induced by *X*, the crossing values between elements $\pi _i$ and $\pi _{i\pmod {n}+1}$ impacted by $\alpha$ are different from zero, which implies that $e = \{\pi _i, \pi _{i\pmod {n}+1}\} \in E(G^{X}_{\pi })$. By definition, in $G^{X'}_{\pi '}$ this edge either decreases its weight by one or is removed, so $cc(G^{X'}_{\pi '}) \ge cc(G^X_{\pi })$.

Suppose first $cc(G^{X'}_{\pi '}) = cc(G^X_{\pi })$. This means that the SSO applied to $\pi$ leaves the connected component $C_{\alpha }$ to which it is applied in $G^{X}_{\pi }$ unchanged. If the SSO is a 2-reversal (resp. a 2-transposition), two (resp. zero) elements inside $C_{\alpha }$ have changed sign. In both cases, we have that $\Delta {cc^-} = cc^-(G^{X'}_{\pi '}) - cc^-(G^X_{\pi }) = 0$.

Now let us suppose $cc(G^{X'}_{\pi '}) > cc(G^X_{\pi })$. This means that $C_{\alpha }$ has been split into two connected components $C_{1}$ and $C_{2}$, thus $cc(G^{X'}_{\pi '}) = cc(G^X_{\pi })+1$. If the SSO is a 2-transposition, zero elements of $\pi$ have changed sign. If the SSO is a 2-reversal, two elements changed sign such that one element is in $C_{1}$ and the other is in $C_{2}$. In both cases, if $C_{\alpha }$ is odd then $C_{1}$ and $C_{2}$ have distinct parities, and $\Delta {cc^-} = 0$; if $C_{\alpha }$ is even then $C_{1}$ and $C_{2}$ have the same parity, and $\Delta {cc^-} \in \{0, 2\}$. $\square$

At this point, we know by Lemma 1 that a 1-reversal always increases or decreases the number of odd components by one, and by Lemma 2 that a 2-reversal or a 2-transposition induced by *X* can only increase by two or leave the number of odd components unchanged in the cp-graph.

#### **Lemma 3**

*Let* $X \in \mathbb {Z}^n$ *be a VD-vector for* $\pi$ *. If* $cn(X) > 0$ *, it is always possible to find a cyclic SSO* $\alpha$ *induced by X such that* $X'$ *is a VD-vector for* $\pi ' = \pi \cdot \alpha$ *and* $\Delta {cc^-} = cc^-(G^{X'}_{\pi '}) - cc^-(G^X_{\pi }) = 0.$

#### *Proof*

Let $\alpha$ be a swap induced by *X* (recall that by definition, $\alpha$ is either a 2-reversal or a 2-transposition), and let $\pi ' = \pi \cdot \alpha$. Note that, since $\alpha$ is induced by *X*, applying it to $\pi$ necessarily decreases by one unit the weight of an edge from $G^X_{\pi }$ in $G^{X'}_{\pi '}$, thus $cn(X') = cn(X)-1$ and $X' \ne X$.

If $cc(G^{X'}_{\pi '}) = cc(G^X_{\pi })$, then, by Lemma 2, we know that $\Delta {cc^-}=0$ and we are done.

Otherwise, we necessarily have $cc(G^{X'}_{\pi '}) > cc(G^{X}_{\pi })$. As shown in the proof of Lemma 2, if the component $C_{\alpha }$ impacted by $\alpha$ in $G^X_{\pi }$ is odd, we know that $\Delta {cc^-}=0$ and we are done. Now suppose that the component $C_{\alpha }$ impacted by $\alpha$ in $G^X_{\pi }$ is even. Let us consider the two components obtained from $C_{\alpha }$ after $\alpha$ is applied. If both components are even, then trivially $\Delta {cc^-} = 0$ and we are done again. Finally, if both components are odd, then we can replace $\alpha$ by $\alpha '$, where $\alpha '$ (i) acts on the same elements of $\pi$ as $\alpha$, and (ii) is a 2-transposition (resp. a 2-reversal) if $\alpha$ is a 2-reversal (resp. a 2-transposition). Note that $\alpha '$ is induced by *X*, and that applying $\alpha '$ also yields two connected components on $V(C_{\alpha })$. Moreover, since 2-reversals change signs while 2-transpositions do not, the two components obtained in the new cp-graph after $\alpha '$ is applied on $\pi$ are both even. Thus $\Delta {cc^-} = 0$ and $\alpha '$ is the sought SSO. $\square$

#### **Lemma 4**

*Let* $\mathcal {S}$ *be a sequence of cyclic SSOs that sorts a permutation* $\pi$ *, and let* $X \in \mathbb {Z}^n$ *be its associated VD-vector. If* $\mathcal {S}$ *is a minimum-length sequence of all sorting sequences induced by X, then* $\mathcal {S}$ *does not contain SSOs that increase the number of odd components.*

#### *Proof*

We will prove the following: if a sorting sequence $\mathcal {S}$ for $\pi$, of VD-vector *X*, contains a cyclic SSO that increases the number of odd components at some point in $G^X_{\pi }$, then we can always find an alternate sorting sequence $\mathcal {S'}$ for $\pi$, also with associated VD-vector *X*, that contains no cyclic SSO that increases the number of odd components, and such that $|\mathcal {S'}| < |\mathcal {S}|$.

Note that, in order to sort a permutation, we need to end up with a cp-graph with *n* even components. From Lemmas 1 and 2, we have that only 1-reversals can decrease the number of odd components. Then, $\mathcal {S}$ contains at least $cc^-(G^X_{\pi }) 1$-reversals. Suppose that $\mathcal {S}$ is a minimum-length sequence that sorts $\pi$, and that $\mathcal {S}$ has an SSO $\alpha$ that increases the number of odd components.

If $\alpha$ is a 1-reversal, then it is necessarily applied to an even component. Thus, the total number of 1-reversals of $\mathcal {S}$ must be greater than or equal to $cc^-(G^X_{\pi }) + 2$. In that case, let $\mathcal {S'}= \mathcal {S} - \{\alpha ,\alpha '\}$, where $\alpha '$ is a 1-reversal applied to the odd component created by $\alpha$. Note that we apply the same sequence of swaps in $\mathcal {S'}$ and $\mathcal {S}$, so both sequences are induced by *X*.

If $\alpha$ is a 2-reversal (resp. a 2-transposition), then, as shown in the proof of Lemma 2, it is necessarily applied to an even component $C_{\alpha }$, transforming it into two odd components. Thus, the total number of 1-reversals in $\mathcal {S}$ is greater than or equal to $cc^-(G^X_{\pi }) + 2$. Let $\mathcal {S'}$ be the sequence obtained from $\mathcal {S}$ by changing $\alpha$ into the 2-transposition (resp. 2-reversal) acting on the same elements as $\alpha$, and by removing the two 1-reversals applied to the odd components created by $\alpha$. Because $\alpha$ has been transformed into a 2-transposition (resp. a 2-reversal), it now creates two even components from $C_{\alpha }$. Note that we apply the same sequence of swaps in $\mathcal {S'}$ and $\mathcal {S}$ (in this case they differ only at the type of swap but it uses the same pair of elements), so both sequences are induced by *X*.

In the above cases, the new sequence $\mathcal {S'}$ is also a sorting sequence for $\pi$, and of length $|\mathcal {S'}| = |\mathcal {S}| - 2$, a contradiction to the fact that $\mathcal {S}$ is of minimum length. Thus $\mathcal {S}$ does not contain SSOs that increase the number of odd components. $\square$

#### **Lemma 5**

*Given a permutation* $\pi$ *, let* $\mathcal {S}$ *be a sequence of cyclic SSOs that sorts* $\pi$ *, and let* $X \in \mathbb {Z}^n$ *be its associated VD-vector. If* $\mathcal {S}$ *is a minimum-length sequence of all sorting sequences induced by X, then* $\mathcal {S}$ *only uses cyclic SSOs that do not increase the edge weights in* $G^X_{\pi }.$

#### *Proof*

We will prove the following: if a sorting sequence $\mathcal {S}$ for $\pi$, of VD-vector *X*, contains a cyclic SSO that increases the weight of an edge *e* at some point in $G^X_{\pi }$, then we can always find an alternate sorting sequence $\mathcal {S'}$ for $\pi$, also with associated VD-vector *X*, that contains no cyclic SSO that increases the weight of an edge, and such that $|\mathcal {S'}| \le |\mathcal {S}|$.

Suppose, without loss of generality, that a cyclic SSO in $\mathcal {S}$ increases the weight of an edge *e* in the cp-graph, and consider the first such SSO, say $\alpha$. Note that, since 1-reversals do not change the cp-graph, $\alpha$ is necessarily a 2-reversal or a 2-transposition. Note also that since this swap increases the weight of an edge, it is not induced by *X*.

If $\alpha$ is applied to two elements in the same component, then $cc(G^{X'}_{\pi '}) = cc(G^X_{\pi })$ and $cc^-(G^{X'}_{\pi '}) = cc^-(G^X_{\pi })$, with $\pi ' = \pi \cdot \rho$.

Otherwise, $\alpha$ is merging two components, say *A* and *B*, and $cc(G^{X'}_{\pi '}) = cc(G^X_{\pi }) - 1$. If both components are odd, then the resultant component will be even, so $cc^-(G^{X'}_{\pi '}) = cc^-(G^X_{\pi }) - 2$, and $cc^-(G^{X'}_{\pi '}) = cc^-(G^{X}_{\pi })$ otherwise. Since we increase the weight of *e*, then $cn(X') = cn(X)+1$. It follows that $cn(X') + cc^-(G^{X'}_{\pi '}) \ge cn(X) + cc^-(G^X_{\pi }) - 1$.

Let $\alpha '$ be the operation that decreases the weight of *e* at some point during the sorting such that $\pi ''' = \pi ''\cdot \alpha '$, where $\pi ''$ is the permutation with all operations before $\alpha '$ in the sorting sequence applied. By Lemma 3 we have that $\alpha '$ decreases the crossing number by one unit and keeps the same number of odd components so $cn(X''') + cc^{-}(G^{X'''}_{\pi '''}) = cn(X'') + cc^{-}(G^{X''}_{\pi ''}) - 1$. It follows that both $\alpha$ and $\alpha '$ are decreasing the sum of crossing number and odd components by one unit at most.

Let $\mathcal {S}' = \mathcal {S} - \{\alpha ,\alpha '\}$. If $\alpha$ merged two odd components *A* and *B*, we add at the beginning of $\mathcal {S}'$ two 1-reversals: one applied to any element $\pi _i \in A$, the other to any $\pi _j \in B$. As shown in the proof of Lemma 1, each 1-reversal here decreases the number of odd components by exactly one unit and keeps the same crossing number. It follows that the newly built sequence is not longer than $\mathcal {S}$, sorts $\pi$, and uses cyclic SSOs which never increase the weight of edges in the cp-graph, so the only swaps it contains are induced by *X*. $\square$

### A polynomial-time algorithm for Sorting Signed Permutations by SSOs

In this section, we first provide a closed formula for computing the length of a sorting sequence of cyclic SSOs for signed linear permutations based on its associated VD-vector *X*. Then, we provide a polynomial-time algorithm for sorting signed circular permutations by SSOs.

#### **Lemma 6**

*Let* $\mathcal {S}$ *be a minimum-length sequence of cyclic SSOs that sorts a signed linear permutation* $\pi$ *, and let X be its associated VD-vector. Then* $d(\pi ) = cn(X) + cc^-(G^X_{\pi }).$

#### *Proof*

Let us partition $\mathcal {S}$ into two sequences $\mathcal {S}_1$ and $\mathcal {S}_2$ in which $\mathcal {S}_1$ (resp. $\mathcal {S}_2$) contains all 1-reversals (resp. swaps) of $\mathcal {S}$. In addition, since 1-reversals do not modify the order of elements in the permutation, we can assume, without loss of generality, that the swaps of $\mathcal {S}_2$ are applied first. We will show that $|\mathcal {S}_1| = cc^-(G^X_{\pi })$. To see this, suppose that we apply a swap (i.e., a 2-reversal or a 2-transposition) $\alpha$ of $\mathcal {S}_2$ in $\pi$, obtaining a permutation $\pi '$, and let $\mathcal {S'}=\mathcal {S}-\{\alpha \}$, and $X'$ its associated VD-vector. Then, by Lemma 3, we know that $cc^-(G^{X'}_{\pi '})= cc^-(G^X_{\pi })$. In addition, by Lemma 4, the number of odd components is not increased by $\mathcal {S}$ and, by Lemma 1, $cc^-(G^X_{\pi })$ can be reduced only by 1-reversals.

Note that the sum of weights of edges in $G^X_{\pi }$ is $cn(X)$, and, by Lemma 2, applying any cyclic SSO $\alpha \in \mathcal {S}_2$ either increases or decreases this sum by one unit, thus $|\mathcal {S}_2| \ge cn(X)$. By Lemma 5, we can assume that $\mathcal {S}_2$ contains no cyclic SSO that increases the weight of an edge, so it follows that $|\mathcal {S}_2| = cn(X)$. $\square$

Lemma 6 shows us that the problem of sorting a signed permutation $\pi$ by cyclic SSOs is equivalent to the following optimization problem: find a VD-vector $X \in \mathbb {Z}^n$ for $\pi$ which minimizes $cn(X)+cc^-(G^X_{\pi })$.

We will now prove that finding such a VD-vector can be achieved in polynomial time. First, we will introduce Lemma 7, where we show that if a VD-vector *X* has a cp-graph with only one component, then any VD-vector $X'$ with $cn(X') \ge cn(X)$ has $d(\pi ,X') \ge d(\pi ,X)$. Then, in Lemma 8, we will prove that any VD-vector $X^*$ such that $d(\pi ,X^*) = d(\pi )$ necessarily belongs to one of two sets that we will define. Finally, we will show in Theorem 1 that we can find a VD-vector $X^*$ such that $d(\pi ,X^*) = d(\pi )$ in polynomial time.

#### **Lemma 7**

*Consider two VD-vectors X and* $X'$ *of* $\mathbb {Z}^n$ *for a signed linear permutation* $\pi$ *, such that* $cn(X') \ge cn(X)$ *. If* $cc(G^X_{\pi }) = 1$ *, then* $d(\pi ,X') \ge d(\pi ,X).$

#### *Proof*

We know, by Lemma 3, that we can apply $cn(X)$ induced swaps in $\pi$ while keeping $cc^-(G^X_{\pi })$ odd components. Using Lemmas 4 and 5, we have that $d(\pi ,X) = cn(X)+cc^-(G^X_{\pi })$. Using the same argument, we have that $d(\pi ,X') = cn(X')+cc^-(G^{X'}_{\pi })$.

Since $cc(G^X_{\pi }) = 1$, then all elements from $\pi$ are in the same component, and $cc^-(G^X_{\pi }) = 1$ (resp. $cc^-(G^X_{\pi }) = 0$) if there is an odd (resp. even) number of negative elements in $\pi$, and any $X'$ is such that $cc^-(G^{X'}_{\pi }) \ge cc^-(G^X_{\pi })$ (if $\pi$ has an odd number of negative elements then for any VD-vector $X'$ there is at least one odd component in $G^{X'}_{\pi }$).

Since we also have that $cn(X') \ge cn(X)$, it follows that $d(\pi ,X') = cn(X') + cc^-(G^{X'}_{\pi }) \ge cn(X)+cc^-(G^X_{\pi })$, and the lemma follows. $\square$

Let $cn(\pi ) = \min (cn(X) :~X \text{ is } \text{ a } \text{ VD-vector } \text{ for } \pi )$, i.e., $cn(\pi )$ is the minimum crossing number over all VD-vectors for $\pi$.

#### **Lemma 8**

*Let S be the set of all VD-vectors* $X \in \mathbb {Z}^n$ *such that* $cn(X) = cn(\pi )$ *, and let* $S'$ *be the set of all VD-vectors* $X' \not \in S$ *such that* $X' = T_{i,j}(X)$ *, for some* $i,j\in [1..n]$ *with* $i\ne j$ *. Then there exists a VD-vector* $X^*\in S \cup S'$ *such that* $d(\pi ,X) = d(\pi).$

#### *Proof*

Recall that for any VD-vector $X'' \not \in S$ there is a strictly contracting transformation that we can apply. Consider a sequence of strictly contracting transformations applied to $X''$ until we reach a VD-vector $X \in S$. Here we will prove by contradiction that there is always a VD-vector $X'$ in this sequence such that $X' \in S \cup S'$ and $d(X',\pi ) < d(X'',\pi )$, i.e., every VD-vector $X''$ outside $S \cup S'$ has $d(\pi ,X'') > d(\pi )$.

If there is a VD-vector $X \in S$ such that $cc(G^X_{\pi }) = 1$, then, by Lemma 7, for any $X' \not \in S$ such that $cn(X') \ge cn(X)+1$, we have $d(\pi ,X') \ge d(\pi ,X)+1$, and it follows that the VD-vector $X^*$ with $d(\pi ,X^*) = d(\pi )$ is such that $X^* \in S$.

Now suppose that a VD-vector $X \in S$ is such that $cc(G^X_{\pi }) \ge 2$. Since $X \in S$, *X* does not admit a strictly contracting transformation, so, for any two distinct $x_a,x_b \in X$, we have that $x_a - x_b \le n$.

**Fig. 2:**
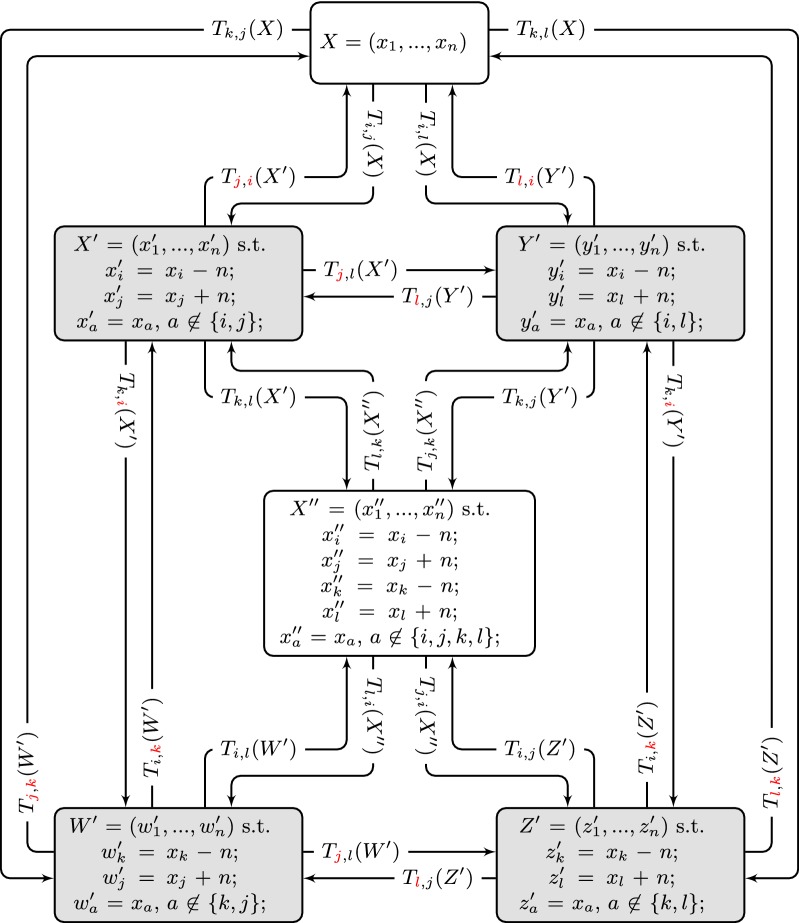
Transformation flow starting with a VD-vector *X*. Note that applying a second transformation at the same positions from the first transformation but reversed results again in *X* (see the transformations where both indices are in red). Note also that every VD-vector obtained from *X* (in gray) can be transformed into two different VD-vectors (that are also obtained from *X*) when we use one of the two indices from the first transformation from *X* (see the transformations from gray VD-vectors where one of the indices is in red). The VD-vector $X''$ can be obtained from the four VD-vectors in gray but not from *X*. Supposing $X \in S$, if all VD-vectors in gray are in $S'$, then $X'' \not \in S \cup S'$. If one (or more) VD-vector in gray is also in *S*, then it follows that $X'' \in S'$

Let $X' \in S'$ be a VD-vector such that $X' = T_{i,j}(X)$ for some $1\le i\ne j\le n$, and let $X''$ be a VD-vector such that $X'' = T_{k,l}(X')$ for some $1\le k\ne l\le n$. As we can see in Fig. 2, if $k = j$ and $l=i$ then $X'' = X$ and $X'' \in S$. If $k = j$ (resp. $l=i$), then $X'' = T_{i,l}(X)$ (resp. $X'' = T_{k,j}(X)$) and $X'' \in S'$.

Suppose now that $X \in S$, $X' \in S'$, and $X'' = T_{k,l}(X')$ is such that $X'' \not \in S \cup S'$. Since $X'' \not \in S \cup S'$, then $i \not \in \{k,l\}$ and $j \not \in \{k,l\}$, and we have that $x'_k = x_k$ and $x'_l = x_l$. This means that, as shown in Fig. 2, $X''$ can be obtained by four different transformations of VD-vectors from $S'$: $T_{k,l}(X')$, $T_{k,j}(Y')$, $T_{i,j}(Z')$, and $T_{i,l}(W')$, such that $X' = T_{i,j}(X)$, $Y' = T_{i,l}(X)$, $Z' = T_{k,l}(X)$, and $W' = T_{k,j}(X)$. The VD-vectors $X', Y', Z'$, and $W'$ are in $S'$, and we say they are *adjacent* to $X''$.

Recall that, since $X \in S$ and $X'' \not \in S \cup S'$, we have that $x_i - x_j < n$, $x_i - x_l < n$, $x_k - x_j < n$, and $x_k - x_l < n$, otherwise at least one VD-vector between $W'$, $X'$, $Y'$, and $Z'$ would be generated by a contracting transformation and is in *S*, and as a consequence $X'' \in S'$. It follows that $cn(X'')> cn(X') > cn(X)$.

Let us suppose that for any VD-vector $V\in S'$ adjacent to $X''$ we have $d(\pi ,X'')<d(\pi ,V)$. Using Lemma 7 we have that values $cc(G^{X'}_{\pi })$, $cc(G^{Y'}_{\pi })$, $cc(G^{Z'}_{\pi })$, and $cc(G^{W'}_{\pi })$ must be strictly greater than 1, otherwise $d(\pi ,X'') \ge d(\pi ,V)$. This observation implies that values $c_{ij}(X)$, $c_{il}(X)$, $c_{kj}(X)$, and $c_{kl}(X)$ are all equal to 1 (recall that, by definition of transformation, if $X' = T_{i,j}(X)$, then $c_{ij}(X') = c_{ij}(X)-2$), otherwise, in at least one of the four VD-vectors in $S'$ adjacent to $X''$ there is a crossing value (in absolute value) greater than one, and, by Property 4, the corresponding cp-graph would have only one connected component.

Hence, we conclude that the four elements $\pi _i,\pi _j,\pi _k$, and $\pi _l$ are in the same component in $G^X_{\pi }$. For the same reason, we have that $x_i,x_k > 0$ and $x_j,x_l < 0$, otherwise at least one of the four VD-vectors in $S'$ contains a displacement value involved in the transformation whose absolute value is greater than or equal to *n* (recall that, by definition of transformation, if $X' = T_{i,j}(X)$, then $x'_{i} = x_i - n$ and $x'_j = x_j + n$), which implies, by Property 2, that this VD-vector has a crossing value with absolute value greater than 1 and, by Property 4, that the cp-graph of this VD-vector has only one component.

**Fig. 3:**
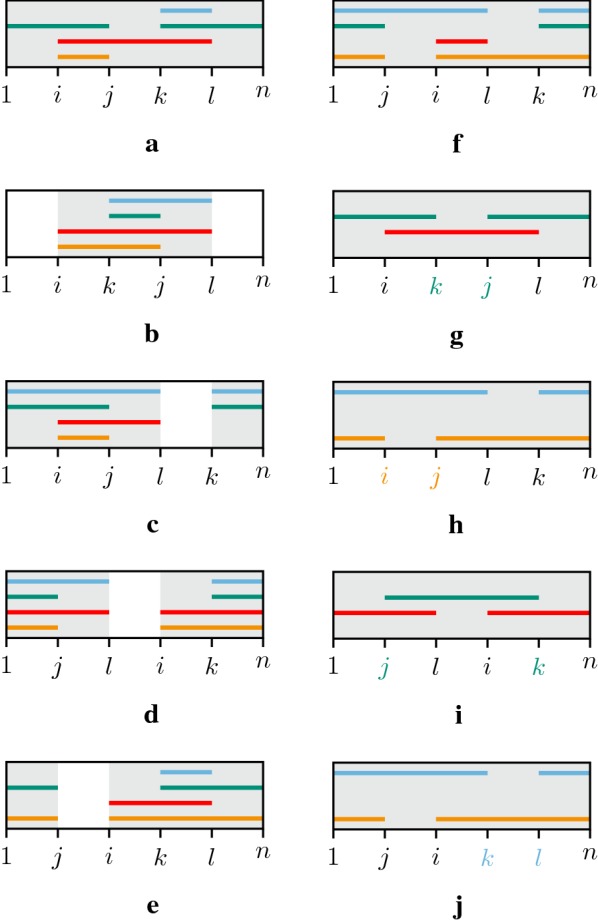
**a**–**f** The six possible configurations for VD-vector $X \in S$, which we call $X^a, X^b, X^c, X^d, X^e, X^f$. For VD-vectors $X^a$ and $X^f$, the union of intervals, highlighted in gray, contains all the elements, thus $cc(G^{X^a}_{\pi }) = cc(G^{X^f}_{\pi }) = 1$. For VD-vectors $X^b$ to $X^e$, the union of intervals does not necessarily contain all elements (cf. white regions), but for each of these configurations there exists a VD-vector $X' \in S'$ obtained by transforming *X*, shown in **g**–**j**, in which the union of intervals, highlighted in gray, also contains all elements, i.e., $cc(G^{X'}_{\pi }) = 1$. Note that in **g** $X' = T_{k,j}(X^b)$, in **h** $X' = T_{i,j}(X^c)$, in **i** $X' = T_{k,j}(X^d)$, and in **j** $X' = T_{k,l}(X^e)$

Now we argue that $X''$ cannot be a vector such that $d(\pi ,X'') = d(\pi )$. Note that since $c_{ij}(X)=c_{il}(X)=c_{kj}(X)=c_{kl}(X) = 1$ we know, by Property 3, that all elements $\pi _a$ with $a \in [i..j] \cup [i..l] \cup [k..j] \cup [k..l]$ must be in the same component. Suppose, without loss of generality, that $i < k$ and $j < l$. We show in Fig. 3 all the possible configurations for these intervals, depending on their relative positions. We can see that we always have either (i) a VD-vector *X* with only one component, so by Lemma 7 $d(\pi ,X) \le cn(X'')$ (Fig. 3a–f), or (ii) a VD-vector $X'$ obtained from *X* with only one component, so by Lemma 7 $d(\pi ,X') \le cn(X'')$ (Fig. 3g–j), and it follows that $d(\pi ,X'') > d(\pi )$. Thus the VD-vector $X^*$ with $d(\pi ,X^*) = d(\pi )$ necessarily satisfies $X^* \in S \cup S'$. $\square$

#### **Theorem 1**

*Finding a VD-vector X for* $\pi$ *that minimizes* $cn(X) + cc^-(G^X_{\pi })$ *can be achieved in polynomial time, and thus sorting signed linear permutations by cyclic SSOs is in* $\mathsf {P}.$

#### *Proof*

Given a permutation $\pi$, we first compute a VD-vector *X* (lines 1–3 of Algorithm 1), then iteratively apply strictly contracting transformations $T_{i,j}(x)$ (lines 4–7) until none exists.

Now let *S* be the set with all VD-vectors *X* such that $cn(X) = cn(\pi )$. Jerrum [13] proved that (i) when no further strictly contracting transformations can be performed on a VD-vector *X*, we have that $cn(X) = cn(\pi )$; (ii) for any two VD-vectors *X* and $X'$ such that $cn(X) = cn(X') = cn(\pi )$, we can go from *X* to $X'$ by a sequence of contracting transformations, i.e., we do not need to go through a VD-vector that is not in *S*. The above properties are in the context of VD-vectors for unsigned permutations, but since VD-vectors for signed permutations do not take into account the signs of the elements we have that (i) and (ii) also apply in our context.

By (i), we have that $X \in S$ (line 9), and, by (ii), we know that we can use *X* to generate the remaining VD-vectors that are also in *S* (lines 10–16). If there is a VD-vector $X \in S$ such that $cc(G^X_{\pi }) = 1$ then, by Lemma 7, we have that for any VD-vector $X' d(\pi ,X') \ge d(\pi ,X)$, so $d(\pi ) = d(\pi ,X)$ and we can just return this value as the sorting distance (line 15). Otherwise, by Lemma 8 the VD-vector $X^*$ with $d(\pi ,X^*) = d(\pi )$ satisfies $X^* \in S \cup S'$, where $S'$ is the set of all VD-vectors obtained by some $T_{i,j}(X)$, with $1\le i\ne j\le n$ and $X \in S$ (instructions in lines 17–21 generate all the VD-vectors of $S'$).

Now we argue that the set of instructions in lines 10–16 of Algorithm 1 either provides all VD-vectors of *S* or at least a VD-vector $X' \in S$ such that $cc(G^{X'}_{\pi }) = 1$. Note that if it provides all VD-vectors of *S*, we just need to generate $S'$ and find the VD-vector $X^*$ with $d(\pi ,X^*) = d(\pi )$. Otherwise, if we have a VD-vector $X' \in S$ such that $cc(G^{X'}_{\pi }) = 1$ then, by Lemma 7, we have that, for any VD-vector $X^*$, $d(\pi ,X^*) \ge d(\pi ,X')$, so $d(\pi ) = d(\pi ,X')$. Arguing by contradiction we show that this set of instructions always leads to one of these two situations.

Suppose that the instructions in lines 10–16 do not provide all VD-vectors in *S* and that, for all VD-vectors *X* provided, we have $cc(G^{X}_{\pi }) > 1$. We have the following facts:

- Since, for any $X \in S$, $cc(G^{X}_{\pi }) > 1$, we must have, by Property 4, that $c_{ij}(X) \in \{-1,0,+1\}$, with $1 \le i \ne j \le n$.
- For a VD-vector $X'$ such that $X' = T_{i,j}(X)$ with $X \in S$, if $X' \in S$, then $cn(X') = cn(X)$, which implies, by Property 1, that $x_i - x_j = n$.
- Since, for any $X \in S$, $\max (X)-\min (X)\le n$, if we have $x_i-x_j=n$ and $x_k-x_l=n$ with $i \ne k$ and $j\ne l$, then $x_i = x_k = \max (X)$ and $x_j = x_l = \min (X)$ (otherwise we have that $x_i-x_l>n$ or $x_k-x_j>n$).
- Note that, by definition of displacement value, $x_k=\sum \nolimits _{l=1}^{n} c_{kl}(X)$. Since, for any $X \in S$, we have $c_{kl}(X) \in \{-1,0,+1\}$, it follows, by definition of cp-graph, that $x_i$ (resp. $x_j$) is located in a component with at least $|x_i|+1$ (resp. $|x_j|+1$) elements on $G^{X}_{\pi }$. If $x_i - x_j = n$ then $x_i$ and $x_j$ must be in the same component, otherwise $G^{X}_{\pi }$ would have at least $|x_i| + |x_j| + 2 > n$ vertices. Besides, this component has at least $\max (|x_i|,|x_j|)+1 \ge \frac{n}{2}+1$ vertices. For the same reason, if we have more than one distinct pair of elements $x_i,x_j$ such that $x_i - x_j = n$, then all these pairs must be in the same component.

Suppose now that we have a VD-vector $X''$ such that $cn(X'') = cn(\pi )$ (which implies that $X'' \in S$), and suppose that $X''$ cannot be obtained by one contracting transformation on *X* (i.e., Algorithm 1 cannot generate $X''$). We will show that if $X''$ requires at least two contracting transformations then *X* admits a contracting transformation using only indices from the first and the second transformation such that the resulting VD-vector $X^*$ has only one component, and, by Lemma 7, $X^*$ already has $d(\pi ,X'') = d(\pi )$.

Let us assume then, without loss of generality, that $X'' = T_{k,l}(T_{i,j}(X))$, where $T_{i,j}$ and $T_{k,l}$ are two distinct contracting transformations (i.e., $x_i - x_j = x_k - x_l = n$ with $i\ne l$ and $j\ne k$). In a similar way as explained in the proof of Lemma 8, this means that the VD-vector $X''$ can be reached by four distinct pairs of transformations (namely $T_{k,l}(T_{i,j}(X))$, $T_{k,j}(T_{i,l}(X))$, $T_{i,l}(T_{k,j}(X))$, and $T_{i,j}(T_{k,l}(X))$). However, by Property 1, and using the above mentioned facts, we can conclude that $x_i = x_k = \max (X)$ and $x_j = x_l = \min (X)$, so these four intermediate VD-vectors between *X* and $X''$ are also in *S*, since these transformations are contracting transformations.

Since these four vectors are generated by Algorithm 1 and are in the set *S*, and since, for any $X \in S$, we assumed that $cc(G^{X}_{\pi }) > 1$, we have, by Property 4, that $c_{ij}(X)=c_{il}(X)=c_{kj}(X)=c_{kl}(X) = 1$. Now we can use Fig. 3 again to show that, with the above properties and whatever the order in which elements $x_i$, $x_j$, $x_k$, and $x_l$ appear, we always have at least one vector $X' \in S$ reachable from *X* by one contracting transformation such that $cc(G^{X'}_{\pi }) = 1$, a contradiction to the fact that *S* has no VD-vector $X'$ such that $cc(G^{X'}_{\pi }) = 1$.

It follows that Algorithm 1 provides either all VD-vectors $X \in S$ or at least a VD-vector $X' \in S$ such that $cc(G^{X'}_{\pi }) = 1$.

Algorithm 1 presents the above mentioned procedure, which consists in finding a VD-vector *X* that minimizes $cn(X) + cc^-(G^X_{\pi })$, the latter value being the sought distance. We now turn to evaluating the computational complexity of Algorithm 1. Our primary goal is to ensure polynomiality of the algorithm, and our analysis can certainly be improved.

The loop in lines 1–2, line 3, and the loop in lines 4–7 run in linear time each. Line 8 takes $O(n^2)$ time to compute $cn(X)$ plus $O(|V|+|E|)=O(n^2)$ time to compute $cc^-(G^{X'}_{\pi })$, resulting in $O(n^2)$ time. Line 9 runs in linear time. The loop in lines 10–16 runs in $O(n^4)$ time: it iterates in $O(n^2)$ and line 12 runs in $O(n^2)$ time. The loop in lines 17–21 runs in $O(n^6)$ time: it iterates in $O(n^4)$ and line 20 runs in $O(n^2)$ time. The overall running time complexity of our algorithm is then $O(n^6)$.

**Fig. 4:**
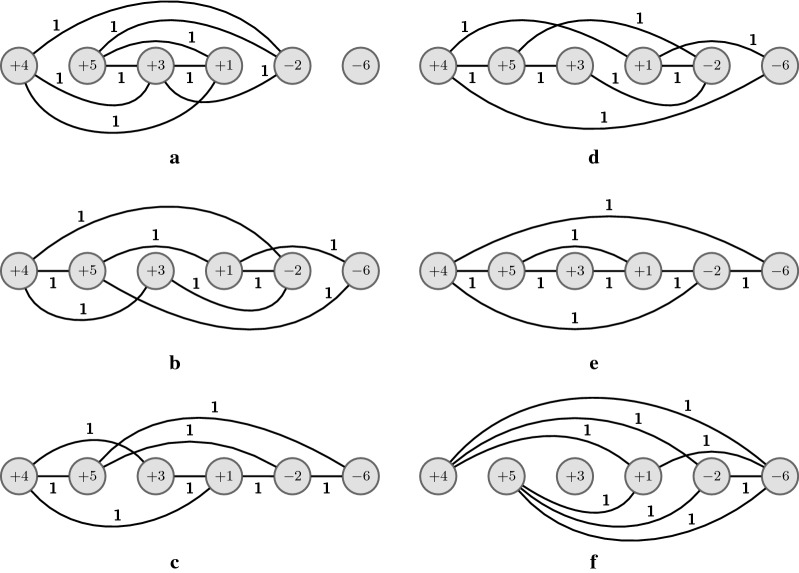
Given $\pi = (+\,4 +5 +3 +1 -2 -6)$, we have in **a** the cp-graph $G^{X^1}_{\pi}$ for $X^1 = (3,3,0,-3,-3,0)$, in **b** the cp-graph $G^{X^2}_{\pi}$ for $X^2 = (3,-3,0,3,-3,0)$, in **c** the cp-graph $G^{X^3}_{\pi}$ for $X^3 = (3,-3,0,-3,3,0)$, in **d** the cp-graph $G^{X^4}_{\pi}$ for $X^4 = (- \, 3,3,0,3,-3,0)$, in **e** the cp-graph $G^{X^5}_{\pi}$ for $X^5 = (- \, 3,3,0,-3,3,0)$, and in **f** the cp-graph $G^{X^6}_{\pi}$ for $X^6 = (- \, 3,-3,0,3,3,0)$. $X^1$ to $X^6$ are the six possible VD-vectors for $\pi$ with minimum crossing number (i.e., $cn(X^i) = cn(\pi ) = 8)$ with $i \in [1..6]$). Note that, by definition, they are in *S*, but Algorithm 1 will not generate all of them. For instance, if the algorithm starts *S* (at line 9) with $X^1$, it will not generate (in loop at lines 10–16) $X^6$ since this VD-vector is reachable from $X^1$ using two transformations but not using only one (note that they differ in exactly four displacement values). As proved in Theorem 1, since Algorithm 1 is not capable of generating all VD-vectors in *S*, then there is at least one VD-vector generated in the path between $X^1$ and $X^6$ with only one component (in this example, the four intermediate VD-vectors $X^2$, $X^3$, $X^4$, and $X^5$ that are generated by Algorithm 1 satisfy this condition)

**Fig. 5:**
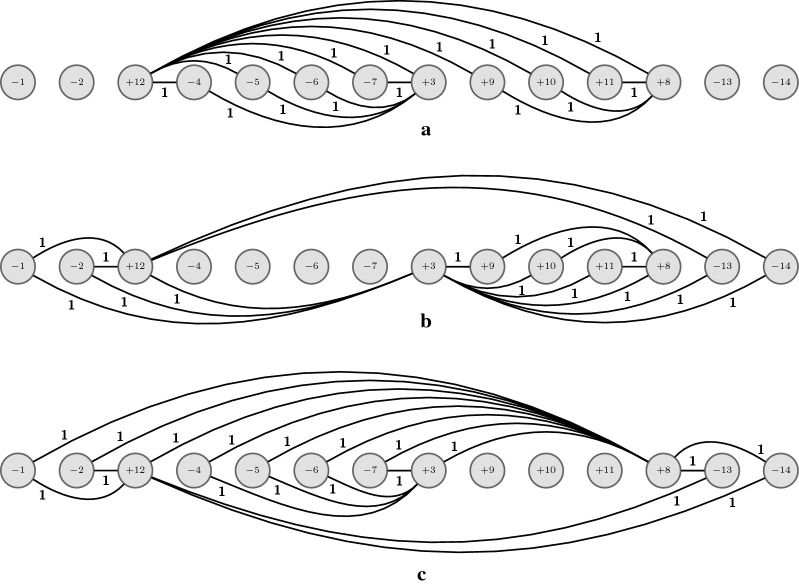
Given $\pi = (-\, 1 -2 +12 -4 -5 -6 -7 +3 +9 +10 +11 +8 -13 -14)$, **a** and **b** show the two cp-graphs $G^{X^1}_{\pi}$ and $G^{X^2}_{\pi}$ for VD-vectors $X^1 = (0,0,9,0,0,0,0,-\,5,0,0,0,-\,4,0,0)$ and $X^2 = (0,0,-\,5,0,0,0,0,9,0,0,0,-\,4,0,0)$. $X^1$ and $X^2$ are the two VD-vectors with minimum crossing number (i.e., $cn(X^1) = cn(X^2) = cn(\pi ) = 16$), so $\{X^1,X^2\} \in S$. Note that $X^2$ (resp. $X^1$) can be obtained from $X^1$ (resp. $X^2$) by $T_{3,8}(X^1)$ (resp. $T_{8,3}(X^2)$), so Algorithm 1 will generate both VD-vectors, starting either with $X^1$ or $X^2$. Note that $cc^-(G^{X^1}_{\pi }) = cc^-(G^{X^2}_{\pi }) = 4$, so $d(\pi ,X^1) = d(\pi ,X^2) = 16+4 = 20$. In **c** we have the cp-graph $G_{\pi}^{X^3}$ for VD-vector $X^3 = (0,0,- \, 5,0,0,0,0,- \, 5,0,0,0,10,0,0)$ with $cn(X^3) = 18 > cn(\pi )$, so $X^3 \not \in S$, but $X^3 \in S'$ since $X^3 = T_{3,12}(X^1) = T_{8,12}(X^2)$. Note that $cc^-(G^{X^3}_{\pi }) = 0$, so $d(\pi ,X^3) = 18$. Among all VD-vectors in $S \cup S'$, $X^3$ is in fact the VD-vector that minimizes the sum and it follows that $d(\pi ) = d(\pi, X^3) = 18$

An example where Algorithm 1 does not generate all VD-vectors in *S* is given in Fig. 4, and an example where Algorithm 1 generates all VD-vectors in $S \cup S'$ is given in Fig. 5. $\square$
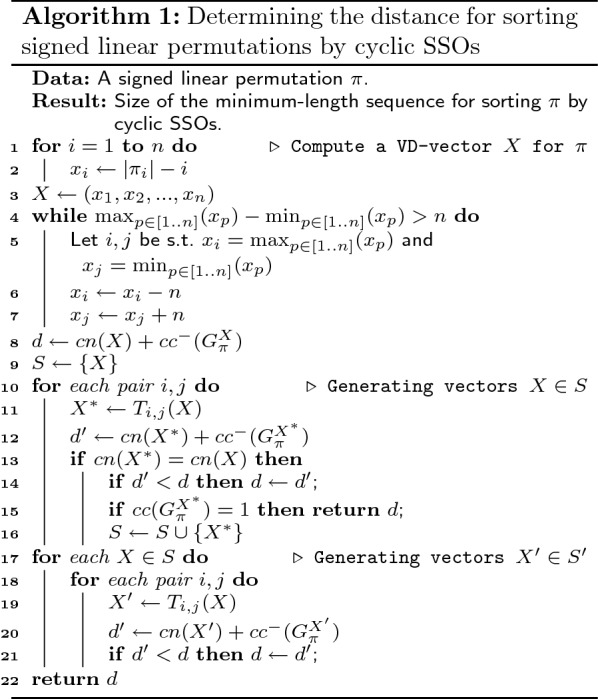

Theorem 1 shows that computing the sorting distance for signed permutations, using cyclic SSOs, is in $\mathsf {P}$. From this, one can easily derive a polynomial-time algorithm for sorting signed *circular* permutations by SSOs: it suffices to cut the circular permutation (*n* different cuts are possible), and to decide which extremity is left and which is right (2 possible cases). Once this is done, we are left with a linear permutation, which can be sorted using cyclic SSOs using Algorithm 1. The sorting distance is the minimum value obtained by Algorithm 1 over the 2*n* possible linear permutations obtained from the circular one. Thus we have the following result.

#### **Theorem 2**

*Sorting signed circular permutations by SSOs is in* $\mathsf {P}.$

### Computing a sorting sequence

Algorithm 1 only returns the length of a minimum-length sorting sequence, not the sequence itself. However, we can easily provide a minimum-length sequence using the cp-graph $G^X_{\pi }$, where $\pi$ is the input signed permutation and *X* is a VD-vector such that $d(\pi ,X) = d(\pi )$. For this, we iteratively remove each edge from $G^X_{\pi }$ by applying a swap over two adjacent elements connected by an edge, and choosing between a 2-reversal or a 2-transposition so that the number of odd components does not increase, as shown in Lemma 3. When $G^X_{\pi }$ has no more edges, we just need to apply $cc^-(G^X_{\pi }) 1$-reversals over the remaining negative elements.

## Conclusion

In this work, we presented a polynomial-time algorithm for sorting signed circular permutations by SSOs. This solution closes a gap in the literature concerning the use of SSOs to sort linear and circular permutations, considering both signed and unsigned versions. Some theoretical questions concerning SSOs and signed permutations remain open, such as diameter issues: what is the maximum distance over all permutations of size *n*? Another interesting question consists in refining the model by taking into account the sizes of the intergenic regions between genes, as was recently done for the classical DCJ distance [17–19]. In particular, sorting by DCJ becomes $\mathsf {NP}$-hard when intergenic regions are considered, while it is in $\mathsf {P}$ otherwise, and SSOs seem to be very well-suited for a similar study.
